# Perceptual, Not Attentional, Guidance Drives Happy Superiority in Complex Visual Search

**DOI:** 10.3390/bs15020124

**Published:** 2025-01-24

**Authors:** Sjoerd M. Stuit, M. Alejandra Pardo Sanchez, David Terburg

**Affiliations:** 1 Department of Experimental Psychology, Utrecht University, 3584 CS Utrecht, The Netherlands; pardomale@gmail.com (M.A.P.S.); d.terburg@uu.nl (D.T.); 2 Department of Psychiatry and Mental Health, University of Cape Town, Cape Town 7700, South Africa

**Keywords:** emotion, eye tracking, visual search, image features, decoding

## Abstract

Emotional facial expressions are thought to attract attention differentially based on their emotional content. While anger is thought to attract the most attention during visual search, happy superiority effects are reported as well. As multiple studies point out confounds associated with such emotional superiority, further investigation into the underlying mechanisms is required. Here, we tested visual search behaviors when searching for angry faces, happy faces, or either happy or angry faces simultaneously using diverse distractors displaying many other expressions. We teased apart visual search behaviors into attentional and perceptual components using eye-tracking data and subsequently predicted these behaviors using low-level visual features of the distractors. The results show an overall happy superiority effect that can be traced back to the time required to identify distractors and targets. Search behavior is guided by task-based, emotion-specific search templates that are reliably predictable based on the spatial frequency content. Thus, when searching, we employ specific templates that drive attentional as well as perceptual elements of visual search. Only the perceptual elements contribute to happy superiority. In conclusion, we show that template-guided search underlies perceptual, but not attentional, happy superiority in visual search.

## 1. Introduction

Facial expressions provide useful communication signals concerning an individual’s emotional state. As such, displaying emotions is thought to be important for the development of complex social structures (Burrows et al., 2006; Parr et al., 2000). In line with their behavioral relevance, faces with emotional expressions are located faster when searched for and attract and hold more visual attention compared to neutral expressions (Palermo & Rhodes, 2007; Vuilleumier & Schwartz, 2001). However, not all emotional expressions affect observers equally. Specifically, Hansen and Hansen observed search asymmetries between finding happy and angry faces (Hansen & Hansen, 1988). They found that angry expressions are found faster among happy distractors than vice versa. Additionally, among neutral distractors, angry expressions were also detected faster compared to happy expressions among neutral distractors. This finding, referred to as an angry superiority effect, has been widely replicated (Ceccarini & Caudek, 2013; Lobue, 2009; Lundqvist & Öhman, 2005). However, a fair amount of research has also found the opposite effect: emotional superiority for happy facial expressions (Becker et al., 2011; Calvo & Nummenmaa, 2008; Hodsoll et al., 2011; Juth et al., 2005). This inconsistency requires an explanation that, due to the number of replications of both angry and happy superiority effects, does not necessarily demand mutual exclusivity.

Aiming to explain why emotional superiority effects differ between studies, Savage and colleagues (Savage et al., 2013) ran a series of experiments using face images from both the NimStim (Tottenham et al., 2009) and Ekman and Friesen (Ekman & Friesen, 1971) databases. They found both happy and angry emotional superiority effects over multiple experiments and showed that the stimulus set, as well as the specific actors, used determined the direction of the superiority effect. Furthermore, both happy and angry superiority effects remained when faces were presented upside-down (Savage & Lipp, 2015). This suggests that emotional superiority effects are not necessarily related to the emotional expressions per se but must be associated with stimulus properties present in the face images. This explanation is in line with previous research showing that it is the effective contrast of the images, and not the emotional content, that is relevant for attracting attention (Gray et al., 2013; Hedger et al., 2015; Hedger et al., 2019; Webb & Hibbard, 2019; Menzel et al., 2018). Focusing on such basic visual properties, Stuit et al. (2021a) found a happy superiority effect for initial eye movements that was predictable via the spatial frequency and orientation content of the images. Importantly, the happy superiority effect was better explained by the basic visual properties of the stimuli than the emotional content of the expression (e.g., the semantic categorization as, for example, a happy expression). The same conclusion was drawn based on an investigation into the emotional superiority effects related to access to awareness, estimated as the time required to perceive an image while it is continuously being masked (Stuit et al., 2023). Again, the emotional superiority effects were predictable yet could not be explained by the emotional content of the images. Still, predictions of first saccades and access to awareness between two facial expressions ignore the intricacies of the visual search tasks upon which the emotional superiority effects are based.

Taken together, emotional superiority effects have been linked to biased attention but with inconsistent effect directions. Moreover, emotional superiority has been linked to specific image confounds and image properties that are unrelated to emotions (note that certain image properties are directly related to the perception of/responses to (specific) emotional faces (Menzel et al., 2018; Webb, 2021; Webb & Hibbard, 2020)). As such, the mechanisms behind emotional superiority appear poorly understood. Visual search in a larger, not specific to emotional faces, context is thought to be guided by search goals, thus resulting in deliberate and meaningful selection of items (Wolfe, 1994). Therefore, visual search behaviors should be, to an extent, predictable based on the visual features of the to-be-searched items. While the prediction of visual search behavior is traditionally based on saliency and priority mapping of the search display, our focus extends to the isolated content of the items in the search display. Note that emotional superiority suggests that specific properties related to the emotional content should consistently attract attention. In other words, true emotional superiority should be independent from the current search goal. In the current study, we therefore not only test for an emotional superiority effect by comparing search times for angry faces with search times for happy faces, but also add a condition where a target can be either angry or happy. This allows us to assess the effect of the search goal on happy or angry superiority directly. Next, we use eye-tracking data to tease apart search times into components associated with foveal and peripheral visual processing of targets and distractors. Here, we aim to distinguish perceptual processing (foveal vision) from attentional capture (peripheral vision). Finally, we aim to predict and compare these aspects of search behaviors using the low-level visual features (spatial frequencies) present in the distractors. For this, we use modeling approaches focused on finding consistent predictors, or, in other words, finding the search templates, that guide the search behaviors.

## 2. Materials and Methods

### 2.1. Participants

Healthy volunteers (N = 31 (12 males); mean age = 23.6, SD = 5.3) with (corrected to) normal visual acuity participated in this experiment in exchange for course credit or payment. All participants provided written informed consent before the experiment started. Two participants were excluded because they did not finish more than half of the experiment. Two other participants who finished most of the experiment (306 and 256 trials of a total of 384) were kept in the analyses. The resulting dataset thus consisted of 29 participants and 10,930 trials. This study was approved by the local ethical committee of the Faculty of Social and Behavioral Sciences at Utrecht University. Furthermore, this research was conducted according to the principles expressed in the Declaration of Helsinki.

### 2.2. Apparatus

The experiment was programmed in E-Prime 2.0 (Psychology Software Tools, Pittsburgh, PA, USA) and presented on the 23-inch integrated monitor of a Tobii TX300 eye-tracker (Tobii Technology, Danderyd, Sweden) with a resolution of 1920 × 1080 pixels and a refresh rate of 60 Hz. The eye-tracking sampling rate was 300 Hz with ~0.5 degrees accuracy. The participants were comfortably seated at a ~60 cm distance from the monitor and responded to the experiment via a combination of gaze fixation and button presses (spacebar of the computer keyboard). Stable synchronization between the experiment PC and eye-tracker was secured using the E-Prime Extensions for Tobii package (EET v2.0.2.41). Eye-tracking and machine learning analyses were conducted using Matlab 2023a. Statistical models for search behaviors were estimated in R (v4.2.2, R Core Team, Vienna, Austria) using the glmer procedure from the lme4 package (Bates et al., 2015).

### 2.3. Stimuli

For the face stimuli, we used the NimStim face database (Tottenham et al., 2009). Only images with an open mouth were used. To allow for a relatively high degree of variance in the visual properties of the faces, and to aid the subsequent machine learning procedures described below, we created a set of unique combinations of the upper and lower halves of two faces of the same gender and emotion (anger, calm, disgust, fear, neutral, sad, and surprise). To be able to do so, the faces needed to be of the same size and have the same position within the images. Since this is not the case for the NimStim face database (or, to our knowledge, any face database), the face area in each image was first detected using the Viola–Jones face detection algorithm (Viola & Jones, 2001). This area was subsequently extracted and scaled such that each face had the same size in all images, with each face in the same position within the images. Next, the faces were matched on average luminance, blended by multiplying each face with a vertically oriented cosine gradient with opposite polarity for each face, and then summed. After blending, each image was tested automatically, by testing whether the Viola–Jones algorithm could still find a face within the image, and manually to find any anomalies (for example, incorrectly scaled faces that, as a result, show a large background area) in the images. Several actors were excluded in this process based on the failure to produce correctly blended faces. For females, this was actor 12, and for males, actors 32, 40, and 43. This resulted in 254 unique face stimuli for each gender–emotion combination (3556 face stimuli in total). Face images were ~2.5 degrees wide by ~4.5 degrees high.

### 2.4. Visual Search Experiment

We used an interactive gaze-contingency paradigm to record visual search behavior and performance. Each stimulus display contained one target face and seven distractor faces. The target face displayed either an angry or a happy emotion, and the distractor faces displayed the following emotions: calm, neutral, disgust, fear, sad, and surprise (one emotion was randomly picked to be displayed twice for each trial).

The participants’ task was to find the target face, look at it, and press the spacebar as fast as possible. Each trial commenced with a fixation cross in the center of the screen for 1500 ms. After a gaze-contingent check that the participant was correctly fixating their gaze, the stimulus display was presented. If no gaze fixation could be detected, a screen appeared showing the eyes of the participant in relation to the quality of eye tracking and the participant was instructed to adjust their position and restart the fixation cross with a button press. After the trial, gaze-contingent feedback was provided immediately after the participant’s button press by presenting a square around the face that was looked at when the response button was pressed. This square was green when that face was indeed the target face or red when it was a distractor face. If no response was given within 5 s after stimulus display onset, the response was scored as incorrect. Three different search tasks were presented to the participants: ‘Find the angry face’, ‘Find the happy face’, and ‘Find the angry or happy face’. These three task conditions were presented in blocks of 32 trials. The block order was pseudorandom and mirrored, that is, X-Y-Z-X-Y-Z-Z-Y-X-Z-Y-X, where X, Y, and Z were assigned to the three task conditions in a counterbalanced fashion across the participants. Consequently, each participant completed 384 trials with an even spread of the three task conditions across the experiment. Each block started with a self-paced on-screen explanation of the upcoming task condition, and the participants could take a one-minute break in between blocks or proceed immediately if they wanted to. Three blocks including four random trials for each condition preceded the experiment for practice. The practice block order was randomized. To ensure an equal distribution of faces in relation to the initial fixation cross, the stimulus display (a mid-screen rectangle of 28 × 22 degrees) was divided into main quadrants, which again were divided into sub-quadrants, resulting in 16 possible image positions. First, the target was assigned a sub-quadrant such that each position was used equally often within a block. Next, the distractors were equally divided over the other main quadrants and randomly assigned a sub-quadrant within each main quadrant. All faces were positioned in the center of their assigned sub-quadrant with a random jitter (x ± 1 degree, y ± 0.23 degrees). Faces were drawn from a pool (see Stimuli) that included 254 images for each emotion (angry, happy, calm, neutral, disgust, fear, sad, and surprised) and gender (male, female). Faces for each emotion were randomly picked (without replacement) with the restriction that the target gender was equally divided within each block and gender was equally divided within each trial.

### 2.5. Procedure

The participants were invited to the lab, briefed about the experiment, and asked to sign an informed consent form. Next, the eye-tracker was calibrated using the 9-point calibration procedure from EET, immediately followed by instructions, practice trials, and the visual search experiment.

### 2.6. Gaze Fixation Detection

We used a dispersion algorithm to identify gaze fixations in the gaze data. A gaze data point was considered part of a potential gaze fixation when it was located within a circle (vertical/horizontal radius of 2.5 degrees) of the average location of the preceding gaze data points of that potential gaze fixation. A potential gaze fixation was considered a true gaze fixation when its duration exceeded 100 ms and discarded if that was not the case. Eye blinks and invalid gaze data, as defined by the Tobii eye-tracker validity coding system, were excluded from the gaze fixation algorithm.

### 2.7. Visual Search Analysis

Trials with incorrect responses as determined by gaze location were discarded (1213 trials). Reaction time (RT) was defined as the time between the onset of stimulus display presentation and the button press. RTs were subsequently divided into search times (STs) and target identification times (TITs) using the eye-tracking data. STs were defined as the time between the onset of stimulus display presentation and the start of the first gaze fixation on the target face. TITs were defined as the time between the start of the first gaze fixation on the target face and the button press. RTs below 100 ms or more than 2 standard deviations from the individuals’ mean were discarded (356 trials). Subsequently, the same procedure was applied to STs (338 trials) and TITs (760 trials). Lastly, STs were divided into distractor selection (DS, number of saccades needed to reach the target) and distractor rejection time (DRT, mean dwell time on the distractor faces before the target face is reached). For each trial, we also assessed whether or not the first fixation was on the target (FFT). RTs, STs, TITs, DSs, DRTs, and FFTs were entered into 2 × 2 generalized linear mixed models using a gamma distribution and an inverse link function (Poisson distribution and log link function for DSs, and binomial distribution and logit link function for FFTs), with the target emotion (happy, angry) and search task (find specific emotion, find either emotion) as within-factors. Data were clustered by participant with a random intercept across the participants.

### 2.8. Image Feature Extraction

Aiming to relate search behaviors to the image properties of the visual search distractors, all distractor face images were first translated into features describing their image properties. Specifically, their spatial frequency information was extracted using the Protosc toolbox (Stuit, 2021; Stuit et al., 2021b) with its default settings. For the spatial frequency content, the Fourier magnitudes were down-sampled by taking the sum of all values corresponding to a particular spatial frequency and orientation range. We used 24 spatial frequency bands and 16 orientation bands, resulting in 384 values describing the contrast energy in each image. This not only reduced the total number of features from 20,000 to 384 unique features per image but also disrupted the influence of phase information, meaning that features lost their spatial specificity. Note that, for the current set of images used in the experiment, the cross-validation performance in classifying the images based on their emotion label (angry vs. happy vs. neutral vs. calm vs. disgusted vs. fearful vs. sad vs. surprised faces), from males to females and vice versa, averaged 48% correct (maximum chance performance over two (male to female and female to male) 1000-iteration permutation tests: 15.31%, *p* < 0.001). Note that the data were split into males and females since male and female faces were never mixed when creating the current stimulus set, thus avoiding any data leaks and overfitting due to having identical sub-parts of the faces in both the train and test data.

### 2.9. Search Behavior Metrics for Modeling

To test whether search behavior is predictable based on basic image features, the data from all participants and all trials were combined to quantify different search-related behaviors for each individual image used in the experiment. Only distractor images that were fixated on at least once were used in the analyses. Three metrics to quantify search behaviors were used:

First-eye-movement behavior: Number of first eye movements received divided by the number of times the image was presented. Note that for this metric, it could also be argued that all distractor images should be used, but the conclusions are not different when using this approach.

Distractor selection behavior: number of fixations received (excluding the first eye movement) divided by the number of times the image was presented.

Distractor decision behavior: average dwell time.

To allow for better generalization between tasks, all metrics were standardized using z-scoring separately per task. This transformed the metrics into relative values indicating, for example, whether a distractor was selected relatively often or relatively rarely independent of the average number of distractors selected during a given trial. Note that comparisons based on absolute values can be found in the visual search analyses.

### 2.10. Modeling Procedures—Within Conditions

Two modeling approaches were used in concert: a stepwise regression approach and a decoding approach. Note that, for the decoding approach, continuous data were divided into quartiles and labeled accordingly. Regression and decoding were combined since stepwise regression is more sensitive to linear relationships, while a decoding approach, given the division into quartiles, is more sensitive to classifying distractors as scoring relatively low or high on a given metric. The procedure used 10 cross-validation iterations. In each iteration, 1/3 of the data were used as holdout data and the rest were used for training. First, a stepwise regression model was trained using 384 Fourier features. On average, the procedure selected 17 features from which to predict outcomes based on the features in the holdout data. These predicted scores were then correlated (Spearman) with the true scores from the holdout data. To estimate chance performance, the same correlation method was used but the true scores were first shuffled, rendering them meaningless. The procedure was repeated 1000 times to generate a distribution of rho values based on chance. For the second approach, features selected via a stepwise regression were used to train a linear discriminant analysis, a classification method used to find a linear combination of features, to predict the quartile to which a distractor image belongs on a given search behavior metric. As with the previous approach, the trained model was applied to the holdout data to extract the cross-validated performance, and a 10,000-sample chance distribution was estimated based on the shuffled holdout data. Based on this chance distribution, test performances were converted to z-scores, which were then converted to *p*-values.

### 2.11. Modeling Procedures—Between Conditions

For cross-fitting and cross-decoding, the averaged intercepts and averaged betas, as estimated over 10 iterations using the stepwise regression procedure describe above, were used to cross-validate scores between task conditions. Specifically, the betas and intercepts estimated from one condition, for example, find angry, were averaged and used in a multiple regression approach to predict scores based on the image features in another condition, for example, find happy. These predicted scores were then correlated with the observed data for that condition (e.g., find happy). Significant performance indicates that behaviors have a similar relationship to the image features in both conditions. Next, the features selected in more than 1 iteration of the stepwise regression procedure were used to train a linear discriminant analysis to predict the quartile to which a distractor image belongs on a given search behavior metric given the data from one condition (e.g., find angry) and applied to predict the quartile based on image features from another condition (e.g., find happy). The predicted quartiles were tested against the true assigned quartiles from the latter condition (e.g., find happy). For both the correlations and decoding performances, the 10,000-sample chance distribution was estimated based on the shuffled data (in the current example, this would be the shuffled find happy data). As for the fitting procedure described above, using this chance distribution, test performances were converted to z-scores, which were then converted to *p*-values.

## 3. Results

### 3.1. Reaction Times

**Figure 1:**
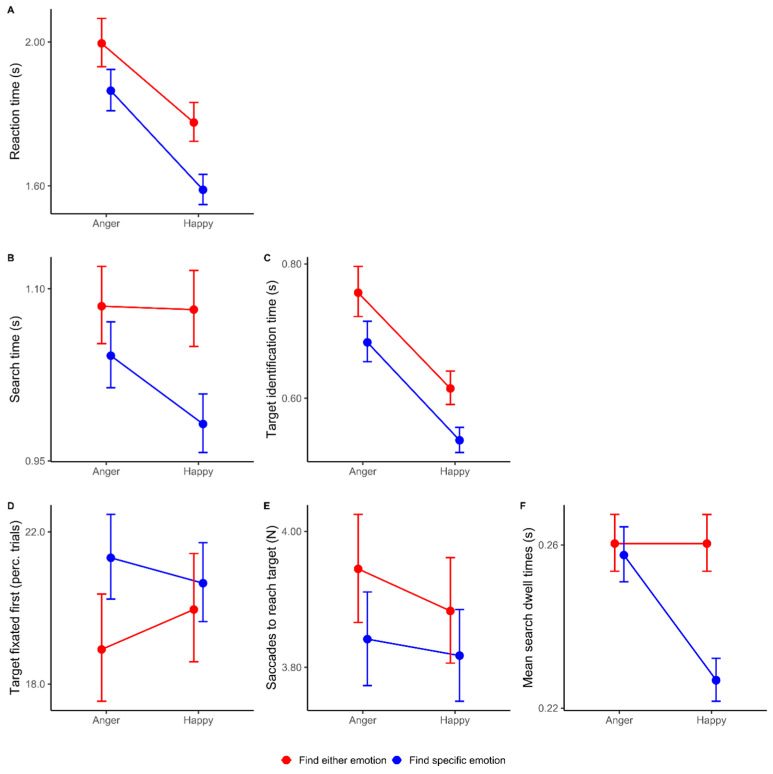
Visual search behavioral results. (**A**) Reaction times. (**B**,**C**) Search times and target identification times, respectively. (**D**–**F**) Percentage of trials where the first fixation was on the target, distractor selection during search, and average dwell times on the distractors, respectively. Red shows the results when the task is to find either emotion, and blue when the task is to find a specific emotion. All values are back-transformed estimated marginal means and standard errors from the associated generalized linear models. These data show clear happy over angry superiority in visual search (**A**) which is due to the time needed to identify (foveal processing of) the target (**C**) and the time needed to discard (foveal processing of) a distractor when searching for a single target (**F**).

As can be seen in Table 1, the RTs show significant main effects for emotion and task (*p* < 0.001). As displayed in Figure 1A, these are due to faster reactions when the task is to find a specific emotion compared to the find-both condition and faster reactions when the target emotion is happy. The interaction of emotion and task is also significant (*p* = 0.002), indicating that the emotion effect is stronger when the task is to find a specific emotion.

### 3.2. Search and Target Identification Times

When we divide the RTs into search times (STs) and target identification times (TITs), we see (Table 1) that the STs show significant main effects for emotion (*p* = 0.029) and task (*p* < 0.001), as well as a significant interaction (*p* = 0.045). As shown in Figure 1B, these effects are due to faster reactions when the task is to find a specific emotion, but only when the target emotion is happy. The TITs show a similar pattern to the RTs, with significant main effects for emotion and task (*p*s < 0.001) due to faster reactions when the task is to find a specific emotion and faster reactions when the target emotion is happy, as well as a significant interaction (*p* < 0.001), indicating that the emotion effect is stronger when the task is to find a specific emotion (see Figure 1C).

### 3.3. Dwell Times, First Fixations, and Number of Saccades During Search

When we divide the STs into the number of saccades on distractors prior to reaching the target (DSs) and distraction rejection times (DSTs), we see (Table 2) that the DSTs show a similar pattern to the STs. The DSTs show significant main effects for emotion and task, as well as a significant interaction (*p*s < 0.001). As displayed in Figure 1F, these are due to faster distractor rejections when the task is to find a specific emotion, but only when the target emotion is happy. The FFTs and DSs are not significantly explained by the factors emotion and task (all *p*s > 0.05, see Figure 1D,E).

### 3.4. Reaction Time Summary

In sum, in the overall reaction times, we see a clear happy over angry superiority as happy faces are found faster than angry faces. This is particularly the case when the task is specifically to find the happy target face. Furthermore, this effect can be largely attributed to the time that is needed to identify the target. Nonetheless, in the search period before that, we still see that happy faces are found faster, but this is only the case when the task is to specifically find a happy target face. Crucially, we can attribute this effect to the time that is needed to decide that a distractor is not the target, rather than being due to a more efficient first fixation or scanning pattern. Thus, with respect to happy over angry superiority in visual search, we can conclude that this is due to the faster identification of happy target faces and the faster rejection of non-happy distractors.

### 3.5. Decoding Distractor-Related Behaviors

**Figure 2:**
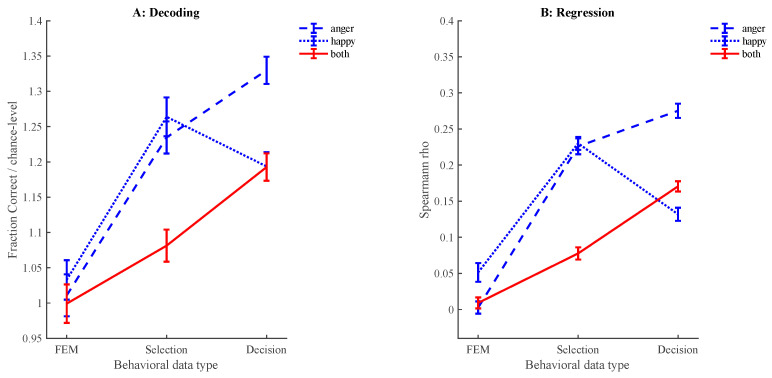
(**A**) shows average fraction correct decoding divided by average chance performance (chance performance equals 1; *y*-axis) for the likelihood of a distractor receiving a first eye-movement (FEM), being selected during search (Selection) and the fixation duration before continuing search (Decision; *x*-axis). All decoding and regression fits are based on the Fourier content of the distractors. The data are separated by task (dotted line: search for angry faces; dashed line: search for happy faces; solid line: search for both happy and angry faces). Error-bars show standard error of the mean of 10-iteration cross validation. (**B**) shows the average spearman r values for 10-iteration cross-validated stepwise regression models. Both figures show that FEM and selection during the search-for-both condition is not predictable, as based on both classification (**A**) and fitting (**B**). Selection during the find angry and happy conditions is predictable above chance, as well as the decision time required in all conditions.

**Figure 3:**
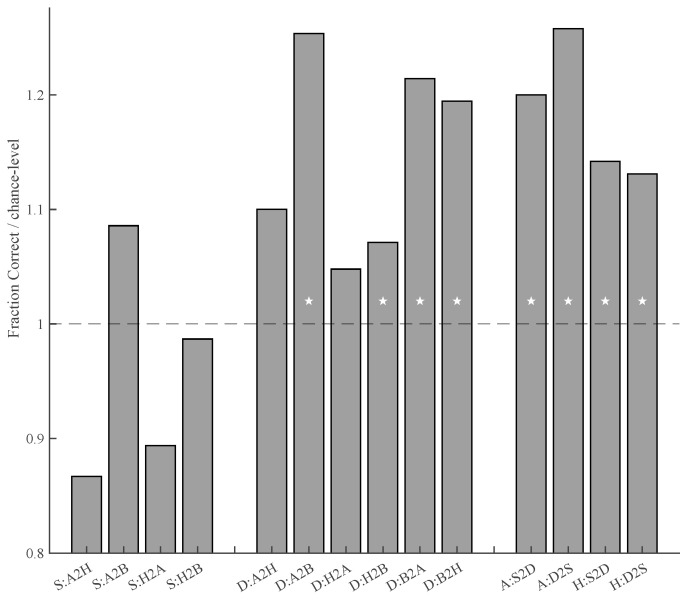
Cross-decoding performances between tasks and search behaviors as average fraction correct decoding divided by average chance performance (chance performance equals 1; *y*-axis). Each bar indicates how well a model based on the data from one condition predicts behavior in another condition. On the *x*-axis are the task and search behavior combinations: ‘S’ refers to selection behaviors, ‘D’ refers to decision behaviors, ‘A’ refers to the find-angry condition, ‘H’ refers to the find-happy condition, and ‘B’ refers to the find-both condition. The ‘2’ indicates the direction of cross-decoding. For example, S:A2H means the model was trained on the selection behaviors of the find-angry condition and applied to the prediction of the selection behaviors in the find-happy condition. White stars indicate significance for both decoding and regression fits (see Appendix A). Note that the models trained on find-happy do not predict find-angry data and vice versa. The results show partial overlap between the models for find-both and the single-target conditions. Cross-decoding for the find-both selection behaviors was not tested as these models did not result in significant predictions within those conditions (Figure 2). Finally, both find-happy and find-angry show significant cross-decoding between selection and decision behaviors in both directions.

All results related to predicting search behaviors, including *p*-values, are shown in Appendix A. All effects noted here refer to both rho values and decoding performance, which are converted to z-scores based on permutation test performances to estimate *p*-values. Predictions are considered significant if both the rho values and decoding performance have a *p*-value < 0.001. The results show that the first eye movement during search is not predictable via the feature content of the distractor images in any of the conditions (Figure 2). Using both regression and classification approaches, the likelihood of the selection of a distractor is predictable in both the find-happy and find-angry conditions (Figure 2). However, these models are task-specific as selection during find-angry does not predict selection during find-happy and vice versa (Figure 3). Selection during the find-both condition is not predictable (Figure 2 and Figure 3). Decision behaviors are predictable for all task conditions, with higher performance for the find-angry condition compared to the other conditions (Figure 2). Cross-decoding decision behaviors between the find-angry and find-happy conditions is not possible (Figure 3). However, cross-decoding is possible from the find-angry to the find-both condition and from the find-happy to the find-both condition, as well as in the opposite direction (find-both to find-angry and find-both to find-happy). Finally, we tested whether the models for selection cross-decode the decision behaviors and vice versa for both the find-happy and find-angry conditions. The results show significant fitting and decoding performances (all *p*s < 0.001).

## 4. Discussion

To understand emotional superiority effects during visual search, we compared search behaviors under various conditions. We established a happy superiority effect in the context of diverse distractors with many different emotions that, like the happy and angry target faces, all featured an open mouth. The results for the reaction time differences show that the overall happy superiority effect is due to the time required to identify distractors and targets when searching for a single target. When searching for both happy and angry simultaneously, we still see an overall emotional superiority effect for happy faces; however, the distractor identification effect disappears and only a target identification effect remains. The results further show that, for single-target search, the selection of distractors and the durations of fixation on distractors are predictable based on their low-level visual characteristics (spatial frequencies). Interestingly, neither selection nor fixation durations in one emotion condition can predict behaviors for the other emotion condition. This suggests that the participants, based on the given task, employ a search template that uniquely contributes to attentional capture and perceptual decision-making processes. Interestingly, these search templates generalize from distractor selection to decision and vice versa, which shows that similar templates are used for attentional capture and decision-making processes. For the dual-target search condition, only distractor fixation durations are predictable by their visual features, suggesting that attention-based search templates do not play a role. Behaviors during single-target search cannot predict behaviors during dual-target search, but single-target fixation durations do predict dual-target search fixations and vice versa, suggesting that subjects employ search templates for both angry and happy faces during the dual-search task. Taken together, our results are in line with guided search (Wolfe, 1994) and thus with task-specific, but not general, biases in attention. In other words, task-based search templates guide visual search for emotional faces, and happy superiority effects arise not from attentional capture for any specific emotional expression, but rather from perceptual decisions about the targets and distractors.

Our results show an emotional superiority effect that is directed towards happy facial expressions. While consistent with numerous studies (Calvo & Nummenmaa, 2008; Hodsoll et al., 2011; Juth et al., 2005), this effect is inconsistent with the large body of literature showing angry superiority effects (Ceccarini & Caudek, 2013; Hansen & Hansen, 1988; Lobue, 2009; Lundqvist & Öhman, 2005). Our results suggest that the effect arises from a difference in the time required to identify happy faces and angry faces, not a difference in attention. Note that this fits well with previously reported advantages in the identification of happy faces (Calder et al., 2000; Calvo, 2008; Palermo & Coltheart, 2004; Terburg et al., 2012; Tottenham et al., 2009), as well as results suggesting that the effect of emotional content on saccades is limited (Stuit et al., 2021a; Webb et al., 2022). So how does this fit with the body of research showing angry superiority effects? First, previously suggested image confounds (Purcell et al., 1996; Purcell & Stewart, 2010) and actor-specific superiority effects (Savage et al., 2013) may have played less of a role due to the manipulation of the face images, where all were scaled to the same size, identities were mixed, and non-facial information such as hair was cropped out of the images. Moreover, our results add an additional explanation for angry superiority effects when using search asymmetries between happy and angry target distractor combinations (Hansen & Hansen, 1988). Specifically, when distractors show happy expressions, and happy expressions are identified faster than angry faces, the net effect is that trials using happy distractors are completed faster than trials using angry distractors, even if the number of items investigated is the same. Note that we also see this in our eye-tracking data (Figure 1F). Therefore, a role for attentional biases is not required for emotional superiority effects in search asymmetry designs, but eye-tracking methodology could be used to effectively tease them apart. Consistent with this is that the models for predicting distractor selection based on feature extraction are task-specific and thus not compatible with a constant, universal, attentional bias throughout search.

Therefore, we argue that attentional biases towards either happy or angry faces do not contribute to emotional superiority. Distractor selection is, however, predictable based on spatial frequencies. Interestingly, these effects are task-specific and predict distractor identification. This fits well with the idea that different search templates are used when looking for a specific emotion, but also suggests that attentional capture does contribute to search for emotional faces. The behavioral data, however, clearly show that, even if attention might be a task-specific factor in search, this does not contribute to emotional superiority in the present task. Specifically, while we observe a behavioral effect during search (Figure 1B), this ‘search’ effect is not attributable to immediately locating the happy facial expression (Figure 1D) or even the time taken to find the target (Figure 1E). Instead, it can be explained by the duration of fixation on distractors. The data suggest that when the target is a happy expression, it is easier to identify a distractor as ‘not the target’. With this in mind, it is important to note that the design of our task features a wide and diverse collection of distractors, which we chose by design to be able to use their features in our feature extraction modeling approach. This relatively large number of features present in each stimulus display also introduces additional task complexity. It might therefore be the case that, in our design, perceptual processing has gained the upper hand compared to attentional capture. Indeed, when relying on perceptual processing, for instance, during emotion recognition, happy faces are easier to process compared to angry faces (Calder et al., 2000; Calvo, 2008; Palermo & Coltheart, 2004; Terburg et al., 2012; Tottenham et al., 2009). In light of our observation that attentional processes do play a role during search, it is therefore possible that these processes can contribute to emotional superiority in designs that are less complex. Indeed, Öhman and colleagues put forward the argument that angry superiority particularly comes into play in simple search designs with similar distractors and/or the use of schematic faces (Öhman et al., 2012). Future studies could therefore focus on the methodology to make these simpler designs suitable for feature extraction algorithms as used in the present study in order to see whether attention-based search templates can also contribute to emotional superiority.

Finally, the find-both condition of our experiment is an especially interesting case regarding search templates. When looking at the distractor dwell times (i.e., distractor rejection), this condition shows that happy superiority is search-task-specific as it does not generalize to the find-both condition. Our feature extraction results furthermore suggest that the participants employed their angry face template when trying to find either happy or angry faces. Given that happy targets were identified faster, it might thus be the case that the participants divided their resources to focus on the more difficult task and thus effectively searched for angry faces in the find-both condition. Furthermore, while our template analyses focus on Fourier contrasts, these contrasts are affected by the structural components of the images. Given the task-specific nature of the templates, the search behaviors are likely based on the expected structural properties of happy and angry faces.

## 5. Conclusions

In conclusion, here, we show that guided search underlies happy superiority in visual search. When looking for a target emotion, participants employ specific spatial frequency-based search templates that drive attentional as well as perceptual elements of visual search, but only the perceptual elements contribute to happy superiority.

## Figures and Tables

**Table 1:** Results from the statistical models predicting Reaction Times, Search Times, and Target Identification Times.

	Reaction Times			Search Times			Target Identification Times		
Predictors	Estimates	CI	*p*	Estimates	CI	*p*	Estimates	CI	*p*
(Intercept)	1.746	1.692–1.802	<0.001	2.601	2.478–2.730	<0.001	4.797	4.231–5.439	<0.001
TargetEmotion	0.962	0.957–0.967	<0.001	0.985	0.972–0.998	0.029	0.839	0.828–0.850	<0.001
SearchTask	1.026	1.021–1.031	<0.001	1.034	1.020–1.048	<0.001	1.099	1.085–1.113	<0.001
TargetEmotion × SearchTask	0.992	0.987–0.997	0.002	0.986	0.973–1.000	0.045	0.978	0.965–0.990	<0.001
Random Effects									
σ^2^	0.17			0.38			0.21		
τ_00_	0.00_Subject_			0.01_Subject_			0.03_Subject_		
ICC	0.01			0.02			0.13		
N (subjects)	29			29			29		
Observations	9361			9023			8601		
Marginal R^2^/Conditional R^2^	0.013/0.023			0.004/0.021			0.148/0.256		

**Table 2:** Results from the statistical models predicting Dwell Times, Number of Saccades During Search and First Fixations on Target.

	Average Dwell Times			Number of Saccades			First Fixation on Target		
Predictors	Estimates	CI	*p*	Incidence Rate Ratios	CI	*p*	Odds Ratios	CI	*p*
(Intercept)	54.253	44.531–66.098	<0.001	3.871	3.750–3.997	<0.001	0.202	0.188–0.217	<0.001
TargetEmotion	0.877	0.859–0.895	<0.001	1.006	0.995–1.017	0.323	0.994	0.938–1.054	0.852
SearchTask	1.165	1.141–1.189	<0.001	0.989	0.978–1.000	0.052	1.039	0.980–1.102	0.195
TargetEmotion × SearchTask	0.877	0.859–0.895	<0.001	0.998	0.987–1.009	0.673	1.022	0.964–1.083	0.47
Random Effects									
σ^2^	0.06			0.23			3.29		
τ_00_	0.04_Subject_			0.01_Subject_			0.01_Subject_		
ICC	0.4			0.03			0		
N (subjects)	29			29			29		
Observations	7765			9361			9361		
Marginal R^2^/Conditional R^2^	0.425/0.655			0.001/0.029			0.000/0.004		

## Data Availability

The data presented in this study are available on request from the corresponding author due to the inclusion of the copywrite of the images required to complete the analysis.

## Appendix A

All cross-validated rho values and fractions correct.

The criterion for significance was set to 0.001 for both fitting and decoding.

**Within-Condition Fitting and Decoding: First Eye Movement**
**Find-Angry**
Fit rho: 0.0027352 (z = 0.068518, *p* = 0.94537)
Decoding fraction correct: 0.46613 (z = 0.1435, *p* = 0.88589)
**Find-Happy**
Fit rho: 0.051417 (z = 1.5772, *p* = 0.11475)
Decoding fraction correct: 0.44251 (z = 0.42314, *p* = 0.6722)
**Find-Both**
Fit rho: 0.0091851 (z = 0.2866, *p* = 0.77442)
Decoding fraction correct: 0.46953 (z = −0.0086603, *p* = 0.99309)
**Within-Condition Fitting and Decoding: Selection**
**Find-Angry**
Fit rho: 0.22608 (z = 7.0688, *p* = 1.5632 × 10^−12^)
Decoding fraction correct: 0.31173 (z = 4.2729, *p* = 1.9291 × 10^−5^)
**Find-Happy**
Fit rho: 0.23021 (z = 7.2482, *p* = 4.2233 × 10^−13^)
Decoding fraction correct: 0.33047 (z = 4.4754, *p* = 7.6251 × 10^−6^)
**Find-Both**
Fit rho: 0.077671 (z = 2.4347, *p* = 0.014904)
Decoding fraction correct: 0.27382 (z = 1.329, *p* = 0.18386)
**Within-Condition Fitting and Decoding: Decision**
**Find-Angry**
Fit rho: 0.27531 (z = 8.6684, *p* = 0)
Decoding fraction correct: 0.33337 (z = 6.0369, *p* = 1.5715 × 10^−9^)
**Find-Happy**
Fit rho: 0.1319 (z = 4.1174, *p* = 3.8325 × 10^−5^)
Decoding fraction correct: 0.29879 (z = 3.4874, *p* = 0.00048769)
**Find-Both**
Fit rho: 0.1707 (z = 5.3486, *p* = 8.8661 × 10^−8^)
Decoding fraction correct: 0.29949 (z = 3.4923, *p* = 0.00047892)
**Between-Condition Fitting and Decoding: Selection**
**Selection Find-Angry → Selection Find-Happy**
Fit rho: 0.046484 (z = 2.5295, *p* = 0.011423)
[note: negative z] Decoding fraction correct: 0.22346 (z = −4.4838, *p* = 7.3315 × 10^−6^)
**Selection Find-Happy → Selection Find-Angry**
Fit rho: −0.013719 (z = −0.7361, *p* = 0.46167)
[note: negative z] Decoding fraction correct: 0.22181 (z = −3.3866, *p* = 0.00070756)
**Selection Find-Angry → Selection Find-Both**
Fit rho: −0.036297 (z = −1.9738, *p* = 0.048399)
Decoding fraction correct: 0.273 (z = 2.7443, *p* = 0.0060644)
**Selection Find-Happy → Selection Find-Both**
Fit rho: 0.092746 (z = 5.0317, *p* = 4.8611 × 10^−7^)
Decoding fraction correct: 0.25253 (z = −0.42681, *p* = 0.66952)
**Between-Condition Fitting and Decoding: Decision**
**Decision Find-Angry → Decision Find-Happy**
Fit rho: 0.039962 (z = 2.1824, *p* = 0.029077)
Decoding fraction correct: 0.27536 (z = 2.7857, *p* = 0.0053407)
**Decision Find-Happy → Decision Find-Angry**
Fit rho: 0.028553 (z = 1.5301, *p* = 0.12599)
Decoding fraction correct: 0.25219 (z = 1.0795, *p* = 0.28036)
**Decision Find-Angry → Decision Find-Both**
Fit rho: 0.23671 (z = 13.005, *p* = 0)
Decoding fraction correct: 0.32524 (z = 9.2252, *p* = 0)
**Decision Find-Happy → Decision Find-Both**
Fit rho: 0.065954 (z = 3.5779, *p* = 0.00034637)
Decoding fraction correct: 0.27313 (z = 3.4957, *p* = 0.00047277)
**Decision Find-Both → Decision Find-Angry**
Fit rho: 0.13893 (z = 7.5258, *p* = 5.2403 × 10^−14^)
Decoding fraction correct: 0.29731 (z = 6.2454, *p* = 4.2262 × 10^−10^)
**Decision Find-Both → Decision Find-Happy**
Fit rho: 0.073536 (z = 3.9782, *p* = 6.9447 × 10^−5^)
Decoding fraction correct: 0.29594 (z = 5.5951, *p* = 2.2048 × 10^−8^)
**Between-Task Fitting and Decoding: Selection and Decision**
**Selection Find-Angry → Decision Find-Angry**
Fit rho: 0.15297 (z = 8.2545, *p* = 2.2204 × 10^−16^)
Decoding fraction correct: 0.32457 (z = 6.7606, *p* = 1.3742 × 10^−11^)
**Selection Find-Happy → Decision Find-Happy**
Fit rho: 0.14187 (z = 7.7525, *p* = 9.1038 × 10^−15^)
Decoding fraction correct: 0.30587 (z = 4.8624, *p* = 1.16 × 10^−6^)
**Decision Find-Angry → Selection Find-Angry**
Fit rho: 0.21413 (z = 11.5329, *p* = 0)
Decoding fraction correct: 0.27536 (z = 7.7772, *p* = 7.3275 × 10^−15^)
**Decision Find-Happy → Selection Find-Happy**
Fit rho: 0.17371 (z = 9.4849, *p* = 0)
Decoding fraction correct: 0.26469 (z = 4.4093, *p* = 1.0368 × 10^−5^)
